# Neurodevelopmental trajectories from birth to childhood and their impairments: insights from passive, spontaneous, and locomotor-like movements

**DOI:** 10.3389/fneur.2026.1773387

**Published:** 2026-06-08

**Authors:** Damiana Rubeca, Francesca Sylos-Labini, Irina A. Solopova, Camilla Gizzi, Ambrogio Di Paolo, Arthur Dewolf, Yury Ivanenko, Francesco Lacquaniti

**Affiliations:** 1 Laboratory of Neuromotor Physiology, IRCCS Fondazione Santa Lucia, Rome, Italy; 2 Department of Systems Medicine and Center of Space Biomedicine, University of Rome Tor Vergata, Rome, Italy; 3 Laboratory of Neurobiology of Motor Control, Institute for Information Transmission Problems, Moscow, Russia; 4 Neonatology Unit, Sant’Eugenio Hospital, Rome, Italy; 5 Neonatology Unit, San Giovanni-Addolorata Hospital, Rome, Italy; 6 Laboratory of Biomechanics and Physiology of Locomotion, Institute of NeuroScience, Université Catholique de Louvain, Louvain-la-Neuve, Belgium

**Keywords:** corticospinal maturation, early motor development, interpersonal coordination, muscle tone, neurodevelopmental disorders, surface electromyography

## Abstract

Human motor behaviour emerges from the dynamic interplay between the developing nervous system and the musculoskeletal apparatus, progressing from spontaneous fetal movements to postural control and early locomotion, including interpersonal coordination. Careful analyses of these behaviours may provide insight into the functional integrity of spinal and supraspinal networks, and early detection of abnormalities in high-risk populations such as preterm infants. This review summarizes our knowledge on neuromotor development from birth to early childhood, highlighting how passive, spontaneous, and locomotor-like behaviours reflect the maturation of sensorimotor circuits. We describe the transition from prenatal activity to postnatal movements, and the gradual emergence of coordinated postural and locomotor patterns. Special attention is given to the limitations of observational tools and the growing role of quantitative kinematic, kinetic and electromyographic (EMG) techniques, which enable objective assessment of motor variability, interlimb coordination, neuromuscular activation, primitive locomotor patterns, and interpersonal coordination. By integrating the findings from a rich motor repertoire, such as the assessment of General Movements, with quantitative neurophysiological findings, deviations from typical behaviour can reveal early dysfunction of sensorimotor pathways, and predict later motor impairments such as Cerebral Palsy. Combining these perspectives moves toward more accurate and timely identification of infants at risk, ultimately supporting earlier, individualized intervention strategies.

## 1 Introduction: early motor repertoire and lifespan neural foundations

Human motor behaviour begins prenatally and evolves continuously through neonatal stages into childhood and adolescence, shaping the neural and musculoskeletal systems that support voluntary movement and locomotion (1–3). Early motor activity encompasses spontaneous, reflexive, and postural movements, reflecting the maturation of central nervous system (CNS) circuits (4–6). These early movements not only provide functional output but also generate sensory feedback, which guides the refinement of neural circuits and supports the emergence of coordinated, goal-directed behaviour (3, 7).

Fetal motor behaviour appears as early as 7–8 weeks postmenstrual age, with initial head, trunk, and general flexion movements (3, 8, 9). By 9–12 weeks, isolated limb movements, startles, general movements (GMs), and REM sleep twitches suggest early sensorimotor integration (5, 10). Between 13 and 20 weeks, differentiated behaviours such as hand-to-face movements, sucking, and swallowing emerge, possibly reflecting subcortical and early cortical maturation (9, 11). Later in gestation, movements become more fluent, variable, and responsive to external stimuli (12, 13), influenced by fetal position, maternal posture, circadian rhythms, and behavioural states (14, 15).

After birth, neonates exhibit GMs and primitive reflexes, which serve as sensitive markers of neural integrity. GMs, most comprehensively characterised by Heinz Prechtl and colleagues, are complex, variable, and whole-body movement patterns that occur without external stimulation. They are visible during pregnancy and progress in a clear age-dependent manner after birth, becoming harder to observe after 5 months (4, 16, 17). Primitive reflexes, such as rooting, sucking, Moro, grasp., Babinski, glabella, supporting reaction, and asymmetrical tonic neck reflexes, are elicited by specific sensory stimuli and mediated by subcortical circuits, gradually diminishing as cortical control matures (18–23).

The *stepping reflex* (also known as the *automatic walking* or *primary locomotion reflex*) is one of the most studied reflexes during the first 2 months of life (24). It can be elicited in most neonates when they are held upright with the feet touching a surface, and produce alternating, quasi-rhythmic leg movements resembling a caricature of adult walking. This reflex represents an early expression of subcortical locomotor circuitry, and is often considered a precursor of voluntary walking (1, 25–29).

The progression to independent locomotion relies on the integration of musculoskeletal maturation, neural circuit refinement, postural control, and multisensory feedback. Head control emerges within the first months, followed by sitting, crawling, and cruising, with independent walking typically appearing between 10 and 15 months. All these developmental stages exhibit considerable interindividual variability (30). Experience-dependent sensorimotor learning refines gait and coordination, while spinal networks increasingly interact with cerebellar, basal ganglia, and cortical structures to improve locomotor control (28, 31). Most children achieve locomotion roughly resembling adult gait by 3–4 years, but considerable refinements can be recognized until adolescence (32–35). Adult gait tends to be energy-efficient, organized around stance and swing phases, with coordinated interlimb alternation, quasi-inverted-pendulum dynamics, and muscle synergies controlled by spinal and supraspinal centres (27, 36–38).

All of these stages of motor development, from fetal movements to neonatal activity and eventually independent locomotion, are made possible by the coordinated maturation of neural circuits and the musculoskeletal system. Neural development involves all stages of the sensorimotor loops, from sensory pathways to spinal Central Pattern Generators (CPGs), to brainstem, cerebellum, and cerebrum, which progressively refine voluntary control and sensorimotor integration (37, 39–41). Skeletal muscles also develop early in the foetus, first myotomes appearing at week 5 (42, 43), and are fully functional but immature at birth, with postnatal growth and activity promoting growth, myofibrillar organization, and loss of poly-innervation (44).

Motor patterns from the fetal period through infancy provide a sensitive window into the functional maturation of the hierarchical levels of the CNS. These behaviours are robust indicators of neuromotor integrity, yet detecting deviations that reflect emerging or established pathology remains challenging in clinical practice. Early signs may be subtle, fluctuate with behavioural state, and overlap with physiological variability. This review provides an overview of current insights into neuromotor development from birth through early childhood, emphasizing how spontaneous, passive, and early locomotor-like movements mirror the progressive maturation of sensorimotor networks.

## 2 Diagnostic challenges in the early detection of neuromotor disorders

This section reviews the main diagnostic challenges and summarizes current clinical and quantitative assessment tools used to identify neonates and children at risk for, or presenting with, neuromotor disorders.

### 2.1 Populations at risk for neuromotor disorders

**Figure 1:**
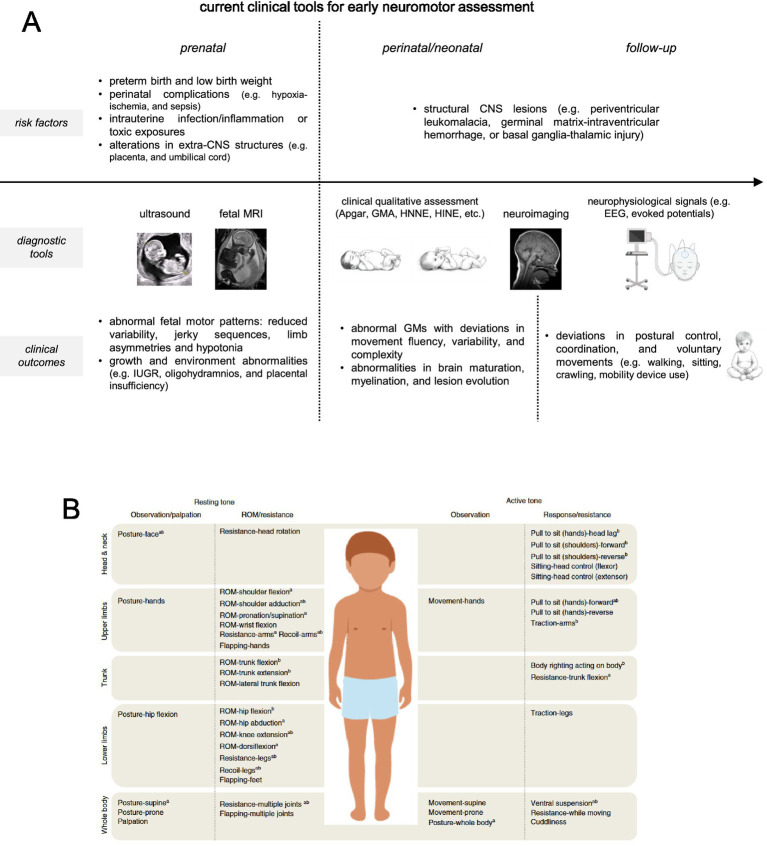
Current clinical tools across early developmental stages. **(A)** Timeline from the prenatal period to the perinatal/neonatal stage and follow-up, illustrating risk factors, diagnostic tools, and clinical outcomes evaluated at each stage. In current clinical practice, prenatal assessment relies primarily on ultrasound and fetal MRI. Neonatal and follow-up evaluations incorporate clinical qualitative assessments (e.g., Apgar score, GMA, HNNE, HINE), neuroimaging, and neurophysiological signals (e.g., EEG, evoked potentials). **(B)** Clinical assessment to evaluate both resting tone and active tone. ROM, range of motion. ^a^Items identified from assessments with ‘strong’ or ‘moderate’ positive evidence in *validity*. ^b^Items identified from assessments with ‘strong’ or ‘moderate’ positive evidence in reliability [reproduced from (106)].

Identifying neonates and infants who may deviate from typical developmental trajectories is central to early neuromotor assessment. Neurodevelopmental trajectories are not only shaped by postnatal adaptation but are deeply influenced by prenatal conditions (Figure 1A). A growing body of evidence highlights the role of maternal health, intrauterine environment, and fetal exposures in modulating early brain development and subsequent motor behaviour. Conditions such as intrauterine growth restriction and placental insufficiency, often stemming from maternal hypertension or preeclampsia, can lead to chronic fetal hypoxia and nutrient deprivation, which significantly alter white matter maturation and cortical connectivity (45–47).

Additional risk factors include congenital malformations, genetic or metabolic disorders, and intrauterine exposure to infection or inflammation (48–50). Furthermore, maternal lifestyle and intended practices, including nutritional status, pharmacological treatments, and exposure to tobacco, alcohol, or illicit substances, are critical determinants. These causative exposures can trigger epigenetic modifications and disrupt fetal synaptogenesis, interfering with the maturation of neural circuits underlying spontaneous and locomotor-like movements (51–54). Increasingly, environmental factors such as air pollution, heavy metals, and endocrine disruptors are also recognized as potent neurotoxins that can induce a pro-inflammatory intrauterine environment, further compounding the risk of motor and cognitive deficits (55, 56). Parallel to these, perinatal complications such as hypoxia-ischemia, stroke, intraventricular hemorrhage, sepsis, or neonatal encephalopathy also represent significant threats to neuromotor integrity (57, 58). Integrating this prenatal history into the interpretation of the early motor repertoire may therefore enhance the clinical relevance of movement-based assessments and improve the identification of infants at risk.

Within this complex clinical landscape, preterm infants, defined as those born before 37 completed weeks of gestation, represent the most frequently monitored population in neuromotor research and clinical practice. Their motor development unfolds outside the womb during a critical period of sensorimotor maturation. Compared to foetuses, preterm neonates are abruptly exposed to gravity, light, handling, variable temperature, and exogenous stimuli that may interfere with the normal development of cortical-subplate circuits and immature descending pathways (59–61). Preterm birth accounts for about 10%–11% of neonates worldwide (over 15 million per year), and remains a major cause of neonatal morbidity and mortality. For instance, in Italy, the most recent data indicate a prevalence of 6.3% (CeDAP Report, 2023). Prematurity, whether spontaneous or medically indicated, is classified as extreme (<28 weeks of gestational age), very (28 to <32 weeks), or moderate-to-late (32–37 weeks). Births occurring before 32 weeks or with weight <1.5 kg are associated with a markedly increased risk of later neuromotor disability (WHO).

Neuromotor disorders in infancy are heterogeneous, arising from alterations in the maturation and functioning of the central and peripheral nervous system (62). These alterations can affect various components of motor control, including muscle tone, strength, coordination, motor planning, and the quality of voluntary and automatic movements (63, 64). Clinically, they may present with weakness, hypotonia or hypertonia, involuntary movements, cramped movements, tremors, or coordination disruption, and can be generalized or focal, affecting specific body regions (65). Main categories of neuromotor disorders include: syndromes characterized by hypotonia [i.e., *hypotonia syndrome* or *floppy infant syndrome* (66, 67)], movement disorders with dystonia, athetosis, or ataxia, (65, 68) and disorders of motor coordination and planning such as *Developmental Coordination Disorder* (DCD) and dyspraxia (69). Complex conditions, including global developmental delay or autism spectrum disorders, may present with qualitative motor alterations (70). *Cerebral Palsy* (CP) is the leading cause of persistent motor disability in childhood, with a prevalence of 1.5–2.5 per 1,000 live births in high-income countries, with markedly higher rates in preterm infants (71–73). CP has been defined as a group of permanent disorders of the development of movement and posture, causing activity limitation, that are attributed to non-progressive disturbances that occurred in the developing fetal or infant brain (74).

CP is due to non-progressive disturbances of the developing brain, motor deficits often accompanied by sensory, cognitive, or communicative impairments (75). CP involves spastic (>80%), dyskinetic, ataxic, or mixed forms depending on the affected CNS regions (76, 77). Clinical signs and symptoms of CP typically emerge and evolve before 2 years of age (72, 78).

### 2.2 Current clinical and instrumental approaches for early neuromotor assessment

Early and accurate diagnosis is challenging but essential, as timely identification enables interventions that leverage the plasticity of the immature nervous system. The diagnostic pathway extends from the prenatal period through infancy, with each stage providing complementary opportunities to detect deviations from typical development (Figure 1A).

Prenatal assessment relies on fetal ultrasound to evaluate gross movements, postural symmetry, and limb activity (10, 79). Reduced complexity, monotonous sequences, or asymmetry may indicate dysfunction in cortical, brainstem, or spinal circuits. In selected high-risk pregnancies, fetal MRI, typically performed after 22 weeks, complements ultrasound by providing detailed evaluation of brain morphology, connectivity, and microstructural integrity (80, 81). Advanced sequences such as diffusion-weighted imaging (DWI), diffusion tensor imaging (DTI), and magnetic resonance spectroscopy (MRS) enable assessment of white matter organization and metabolic maturation (81, 82). Maternal conditions such as infection, systemic inflammation, or chronic hypoxia further contribute to this risk profile, as they can disrupt the maturation of basal ganglia, thalamus, and periventricular white matter, key regions for early motor organization (57, 59, 83).

Postnatally, the Apgar score (84) provides the first standardized assessment of neonatal adaptation, with persistently low scores predicting higher risk of CP and long-term neurodevelopmental impairment (85–87). Behavioural assessment in the neonatal period and follow-up focuses on spontaneous motor activity, reflexes, postural control, and emerging voluntary movements. *Prechtl’s General Movements Assessment* (GMs) evaluates the quality of spontaneous activity, with abnormalities in fluency, complexity, or variability being highly predictive of later neuromotor disorders, including CP (5, 16, 88). Complementary observation of primitive reflexes and structured exams such as the *Hammersmith Neonatal Neurological Examination* (HNNE) allow assessment of tone, symmetry, and brainstem-spinal circuit integrity (89, 90). Between 2 and 24 months of age, the neurological examination progressively shifts toward the evaluation of postural control, coordination, and voluntary movements. The *Hammersmith Infant Neurological Examination* (HINE) is currently considered the gold standard clinical tool in this age range (91, 92). It provides a semi-quantitative score (e.g., *Gross Motor Function Classification System levels*, GMFCS), that correlates with both the severity of motor impairment and later functional classification in CP, based on children’s ability to walk, sit, crawl, and use mobility devices. Developmental and motor performance scales complement neurological examination. The *Test of Infant Motor Performance* (TIMP) (93) is applicable from 34 weeks of gestational age to 4 months post-term and assesses posture and selective control of movement in infants for functional performance in daily life. The *Bayley Scales of Infant and Toddler Development* (BSID-III) assess global motor, cognitive, and language domains (94). The *Alberta Infant Motor Scale* (AIMS) evaluates spontaneous and antigravity movements from 0 to 18 months after birth (95), while the *Peabody Developmental Motor Scales* (PDMS-2) analyze fine and gross motor skills from 0 to 5 years (96). The *Gross Motor Function Measure* (GMFM, 5 months-16 years) (97) provides standardized measures of milestone acquisition and postural control, frequently used in both clinical and research settings.

According to an authoritative, evidence-based review, before 5-months corrected age, the most predictive tools for detecting risk of CP are term-age magnetic resonance imaging (MRI), GMs, and HINE, all with a diagnostic sensitivity of more than 80% (72). However, MRI highlights the location of neurological lesions, but does not describe their functional consequences (98, 99). In addition, a number of cases of CP are negative at MRI, since the impairment is functional but does not have an anatomical counterpart (100–102). On the other hand, GMs and HINE items describe the functional consequences of the disease exclusively on the basis of visible motor behaviour (posture, muscle tone, reflexes, spontaneous and evoked movements, etc.), but are not able to establish which parameters of neuromuscular control of the newborn are altered (5, 91). This is due to the fact that the neuromuscular control is highly redundant: any given movement can be generated by an infinite number of different neuromuscular command signals (103, 104). In addition, there is a great interindividual variability in neuromuscular control strategies in both healthy and sick subjects (58, 105).

Muscle tone is among the most critical features assessed by clinical scales (Figure 1B), with evaluation encompassing both resting and active components. Resting tone consists in the baseline level of muscle contraction, and reflects both neural contributions (sensorimotor spinal and supraspinal circuits) and non-neural components (viscoelastic properties of muscles). In clinical practice, it is typically assessed via observation, palpation, passive range of motion (ROM), or resistance to passive movement, with the popliteal angle (knee extension) and the scarf sign (shoulder adduction) among the most commonly used tests. Active tone, conversely, indicates the child’s ability to respond to environmental stimuli and postural changes. It is usually evaluated through observation, movements opposite to gravity, or resistance to facilitation, primarily using manoeuvres such as pull-to-sit and ventral suspension (106).

In addition to the behavioural assessment, neuroimaging (Figure 1A) enables detection of structural lesions such as periventricular leukomalacia, germinal matrix-intraventricular haemorrhages, cerebellar haemorrhages or basal ganglia-thalamic injuries, which are strongly associated with spastic or dyskinetic CP (107, 108). Longitudinal MRI allows reconstruction of major motor tracts and correlation between white matter microstructure and functional outcomes (109, 110). Functional imaging modalities, near-infrared spectroscopy (NIRS) and functional MRI (fMRI), provide insight into cortical activation and early neural reorganization (111, 112). fMRI, while providing high spatial resolution and insights into functional connectivity, is limited by the need for strict immobilization, sensitivity to motion artefacts, and the logistical complexity of performing scans in fragile neonatal populations. For example, the immature hemodynamic response of the neonatal brain can lead to an inverted or dampened *Blood Oxygen Level Dependent* (BOLD) signal, complicating its interpretation (113, 114). These constraints often restrict its use to research settings and highly specialized centres (115).

Neurophysiological assessment complements imaging and behavioural evaluation, monitoring cortical maturation and sensorimotor integration through electroencephalography (EEG), amplitude-integrated EEG (aEEG), and evoked potentials (somatosensory SEPs, motor MEPs) throughout infancy (116, 117). EEG offers excellent temporal resolution and is more feasible at the bedside, yet interpretation in early life requires careful feature selection and consideration of behavioural state, given the significant variability of EEG patterns across different states. In addition, the presence of artefacts may substantially affect signal reliability (118), as these tools are highly sensitive to the electrical noise of the neonatal intensive care unit environment. Consequently, their predictive consistency is often limited by the lack of standardized normative databases, especially for extremely preterm infants, while the rapid, non-linear changes in brain impedance during early development make longitudinal comparisons particularly challenging (119, 120).

Collectively, while technically demanding, these techniques contribute to early detection, prognostic stratification, and monitoring of intervention efficacy.

## 3 Movement-related approaches for assessing early neuromuscular development

This section explores methods that can usefully complement routine clinical examination by assessing how spontaneous, passive, and early locomotor-like behaviours reveal the maturation of sensorimotor circuits. Particular focus is given to multi-muscle activity patterns, since they offer a view into central nervous system function and its alterations in neurodevelopmental disorders.

### 3.1 General movements and kinematic assessments

General Movements (GMs) provide a sensitive window into early neuromotor development, reflecting spontaneous activity of spinal, subcortical, and cortical networks. As noticed above, they are widely used to identify infants at risk for neuromotor disorders. While observational tools, such as Prechtl’s GMA, remain central, quantitative analyses of kinematics offer more objective measures of movement quality.

**Figure 2:**
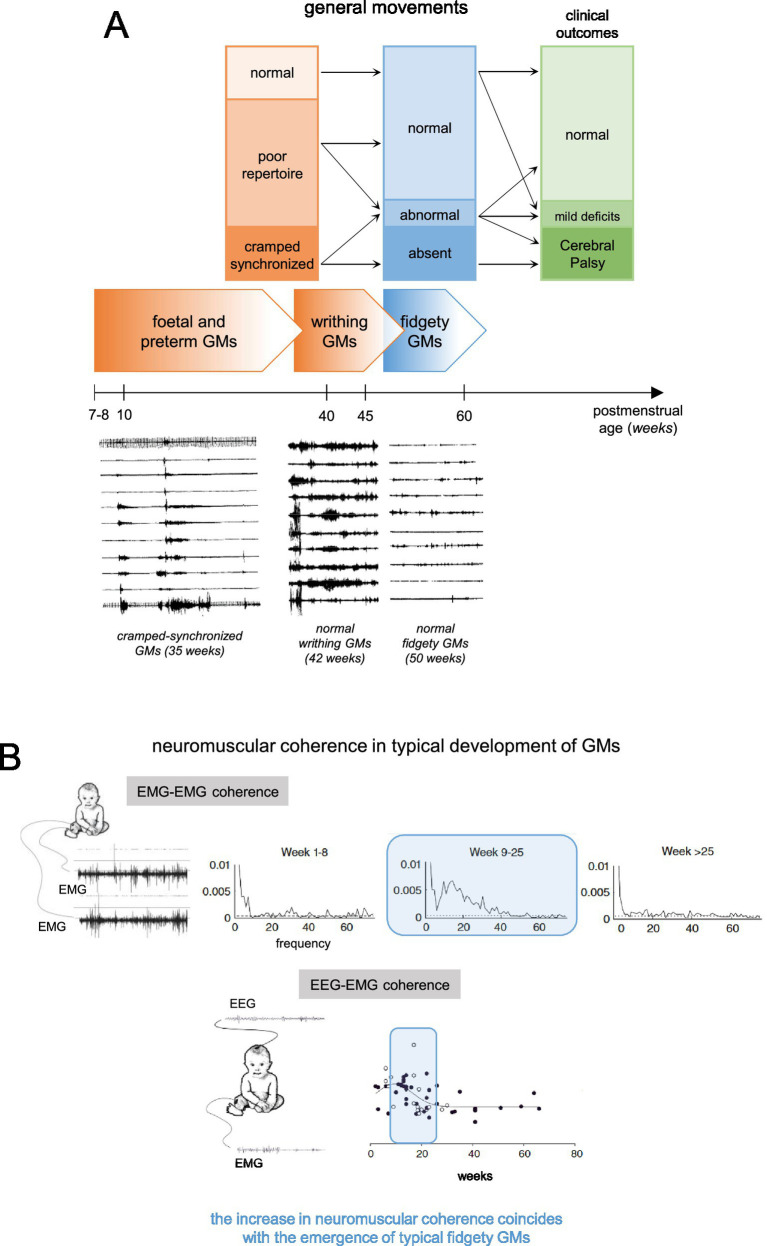
Developmental trajectories of general movements (GMs). **(A)** Diagram illustrates the typical progression of GMs from the fetal period to early postnatal life, showing the transition from writhing to fidgety movements (46–49 weeks postmenstrual age, PMA). Deviations from typical GMs patterns provide early prognostic information: cramped-synchronized movements and absent fidgety movements are associated with a high risk of cerebral palsy, while the characteristics of fidgety movements modulate outcomes in infants who previously exhibited a poor repertoire of writhing movements [redrawn from (121)]. Raw traces of (from left to right): cramped-synchronized GMs [from (137)], normal writhing and normal fidgety GMs [from (136)] are also shown as examples. **(B)** Neuromuscular coherence in typical development of GMs: in the left panel, intramuscular EMG–EMG coherence at different frequencies for the three age groups (1–8, 9–25, and > 25 weeks) from tibialis anterior muscle; in the right panel, logarithm of cortico-muscular EEG–EMG coherence in the 20–40 Hz frequency band plotted against corrected gestational age. The black dots indicate term infants and the white dots indicate preterm infants [adapted from (145)].

GMs are intrinsically generated spontaneous movements, characterized by sequential and variable activation of the arms, hands, legs, feet, neck, and trunk. They begin and end gradually, with speed and intensity that are inherently unpredictable. Prior to term, these movements are classified as *fetal or preterm* GMs. Between 40- and 46–49-weeks postmenstrual age (PMA), they manifest as *writhing movements*, displaying moderate to small amplitudes and slow to moderate velocities (4, 88). Around 46–49 weeks PMA, *fidgety movements* gradually appear, consisting of small, oscillatory motions with moderate speed and variable acceleration, which persist until approximately 6 months of age, when antigravity and more controlled postural movements start to predominate [Figure 2A; (4, 121)].

Kinematic investigations in neonates, initially focused on spontaneous kicking, revealed coordinated joint patterns in healthy full-term infants, with minimal phase lag between flexion and extension and consistent inter-joint coordination (122–124). Other studies have also documented developmental refinements in joint excursions, movement smoothness, and inter-limb coordination, reflecting age-dependent maturation of spontaneous motor activity (8, 125, 126). Subsequent studies extended kinematic analysis to preterm and brain-injured infants, that frequently exhibit altered inter-joint coordination and increased movement variability, which may normalize or persist depending on the clinical context (127–129). Clinically, many preterm infants display *poor repertoire* GMs, characterized by reduced fluency and variability (5, 17). Although the emergence of a poor repertoire does not necessarily indicate a poor prognosis, serial observations are required, as these movements can be followed by normal, abnormal, or absent fidgety patterns. *Cramped-synchronized* GMs, particularly when combined with persistent atypical patterns also in primitive reflexes, are highly predictive of later motor disorders, including CP [Figure 2A; (16, 130, 131)].

The introduction of wearable and video-based technologies has expanded quantitative assessment, allowing longitudinal monitoring in clinical and home settings. Markerless motion capture, accelerometers, and video-derived metrics provide detailed spatial and temporal quantification of GMs. For example, centre-of-mass variability decreases from *writhing* to *fidgety movements*, reflecting the transition from large, unstructured motions to more refined, spatially constrained patterns (132). Composite indices integrating limb acceleration and interlimb jerk correlation have demonstrated high sensitivity and specificity in identifying abnormal GMs, illustrating the clinical potential of low-cost, objective tools (133). Advanced data-driven approaches, including decomposition algorithm (e.g., non-negative matrix factorization) applied to joint kinematics, have further highlighted modular organization underlying apparently variable movements and revealed reduced movement complexity in infants at neuromotor risk (134, 135). While wearable and video-based technologies offer objective quantification, their widespread clinical adoption is currently limited by the need for specialized expertise in data interpretation and the lack of large-scale, standardized paediatric datasets.

### 3.2 Electromyographic analyses

Surface electromyography (sEMG) provides a complementary perspective by capturing the underlying neuromuscular organization of GMs and other motor repertoire beyond its kinematic expression. However, available data are still limited and highly variable across subjects, which challenges the generalization of findings. Early polymyographic studies demonstrated that spontaneous movements in early infancy are produced through broad, overlapping muscle activations, gradually evolving into more temporally precise and spatially differentiated patterns as the nervous system matures (58, 136, 137). Over the first months of life, tonic background activity diminishes, phasic bursts become shorter and more precisely timed, and multi-muscle coordination becomes increasingly structured, reflecting maturation of spinal CPGs and the growing influence of corticospinal pathways. Importantly, sEMG-derived features have been used to classify the quality of GMs and distinguish normal from abnormal neuromotor patterns [Figure 2A; (138)], although the current examples in the literature remain sporadic and require systematic quantitative analyses with standardized performance metrics.

Longitudinal sEMG studies in infants aged one to 6 months have further clarified the emergence of structured multi-muscle patterns and refinement of burst timing, paralleling the behavioural transition from *writhing* to *fidgety* movements, and reflecting ongoing maturation of subcortical and cortical circuits (139). Recent applications of muscle synergy analysis have revealed that spontaneous motor behaviour in early infancy, though highly variable at the surface level, is supported by a modular neuromuscular architecture. Spontaneous kicking recruits multiple, high-dimensional synergies with substantial overlap, whereas early stepping already displays more stereotyped and temporally stable modules that resemble those used during mature locomotion (27, 29, 140). These observations suggest that early spontaneous movements are underpinned by a modular organization of muscle activity, yet the functional significance and the precise relation to emerging structural and neural components require further systematic investigation.

While sEMG provides valuable information on muscle activation and coordination, its interpretation is influenced by electrode placement, signal noise, and the small anthropometric dimensions of infant limbs. This poses a major challenge for signal isolation, as the proximity of small muscle groups increases the risk of *cross-talk*, where electrodes capture overlapping electrical activity from adjacent muscles source (141, 142). Furthermore, the absence of voluntary cooperation in infants prevents the use of traditional normalization techniques, such as *Maximal Voluntary Contraction* (MVC) (143). This lack of a standard physiological reference, combined with limited standardization in neonatal populations, may affect reproducibility and make it difficult to compare muscle activation across different subjects or studies (144). Additional barriers include the high cost of equipment and the limited usability of current sEMG devices for clinicians. Overall, while these methodologies offer important mechanistic insights, their clinical applicability remains constrained by feasibility, standardization, and interpretative challenges. In this context, the assessment of spontaneous motor behaviour represents a complementary and often more accessible approach, with the potential to capture early functional manifestations of underlying neural dysfunction.

### 3.3 Corticomuscular analyses

Recent studies have combined EEG and sEMG to probe corticomuscular and intramuscular coherence as markers of functional corticospinal connectivity in early infancy. Ritterband-Rosenbaum et al. (145) reported a marked increase in both EEG–EMG and EMG–EMG coherence in the 20–40 Hz frequency band in infants aged 9–25 weeks, compared with younger or older infants (Figure 2B). This period coincides with the emergence of fidgety movements, and is characterized by a short-lasting, high-amplitude central peak in EMG–EMG synchronization, suggesting a sensitive window of activity-dependent corticospinal reorganization. Other evidence supports early cortical involvement in motor control: for example, Kanazawa et al. (146) observed descending motor coherence in neonates within the first months of life, revealing positive correlation between EEG–EMG coherence magnitude and postnatal age in the beta frequency band. In paediatric populations with motor disorders, such as dystonia, altered beta-band EEG–EMG coherence has been reported, emphasizing its potential as a biomarker of pathological corticospinal function (147). Together, behavioural observation and quantitative neurophysiology provide a multi-level characterization of early motor function and its developmental trajectory.

### 3.4 Passive movements and early muscle tone assessment

**Figure 3:**
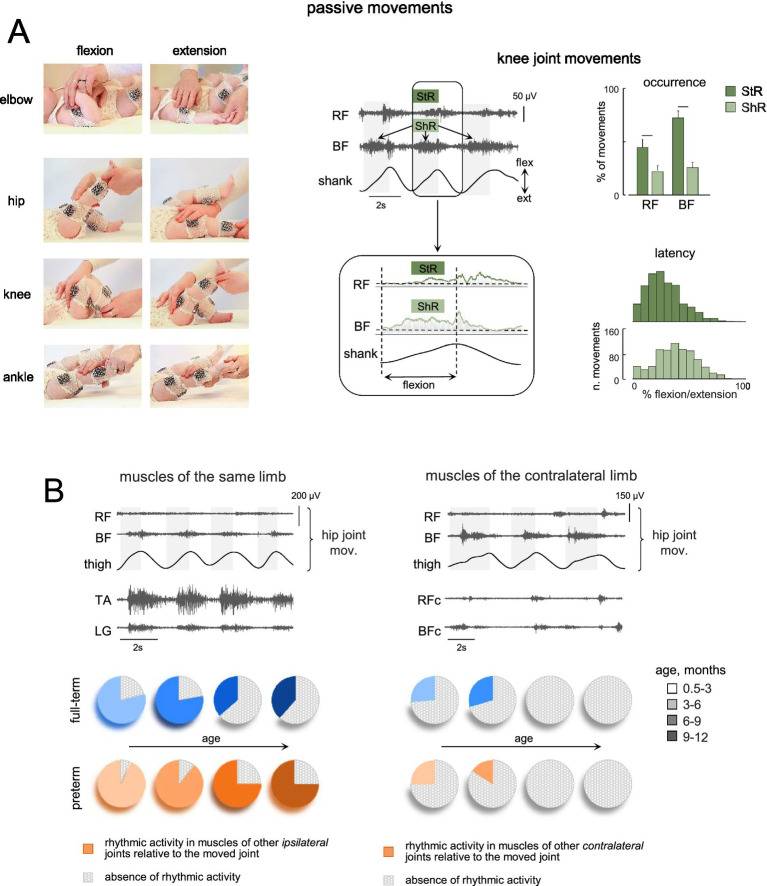
Passive limb movements (PMs) and muscle responses. **(A)** Left: externally imposed PMs (flexion/extension) at elbow, hip, knee, and ankle. Right: example EMG from rectus femoris (RF) and biceps femoris (BF) during knee PMs, showing stretch-related (StR) and shortening-related (ShR) responses. Histogram shows occurrence of StR and ShR, highlighting predominance of StR. Latency distributions (normalized to movement duration) show clear phase-locked onsets (10–50% of cycle), with StR consistently shorter (~0.2 s). **(B)** EMG in muscles of other joints within the ipsilateral and contralateral limbs during hip PM. Pie charts show percentage of infants with rhythmic activity across four age groups (0.5–12 months). Both ipsilateral and contralateral responses decline with age in both full-term and preterm infants, reflecting maturation and progressive independence of limb-specific motor control. RF, rectus femoris; BF, biceps femoris; TA, tibialis anterior; LG, lateral gastrocnemius; c, contralateral. [Adapted from (150, 151)].

Building on the previous discussion of resting and active muscle tone (Figure 1B), passive limb movements provide a more controlled and objective approach to probing the neurophysiological components underlying early tone. Stretch- and shortening-related responses (StR and ShR) may be elicited during externally imposed flexion and extension of the limbs (Figure 3A, left panel). StR reflects resistive responses to muscle stretch, whereas ShR corresponds to compliant responses during muscle shortening, serving as adaptive mechanisms for regulating muscle length and supporting functional motor control (148, 149). Both responses are present from birth in the muscles directly linked to the moved joint, with StR generally occurring more frequently and with earlier onset than ShR (150, 151), and the onset of both typically falling within a consistent portion of the movement cycle (~10%–50%; Figure 3A).

Like spontaneous GMs, passive limb movements generate proprioceptive feedback that continuously informs the developing sensorimotor system, contributing to the fine-tuning of motor responses, the maturation of coordinated motor patterns, and the gradual emergence of functionally appropriate muscle tone (58, 152–154), making them a window into the functional organization of central nervous networks. Transient limb blocks during spontaneous movements, however, do not evoke consistent muscular responses, highlighting the relative independence of limb-specific control and emphasizing that sensory input from actual changes in muscle length is more effective in revealing sensorimotor connectivity (155). StR and ShR frequently co-occur (Figure 3A), reflecting a dynamic regulation of muscle tone and its redistribution among antagonistic muscles (156–159). While clinical assessment of muscle tone typically focuses on stretch responses, providing information on resistance, rigidity, spasticity, or hypotonia, the functional significance of ShR is often overlooked. Importantly, ShR is present from birth as part of the innate repertoire of compliant motor behaviour, observable even in infants as young as 0–3 months (150, 151) when cortical control is still immature and limited (41, 160). This suggests that ShR has a functional role in early adaptive motor development, supporting the gradual acquisition of flexible and coordinated movement.

Beyond the muscles directly involved in the displaced joint, passive limb movements can also elicit regular (rhythmic) activity in other muscles of the same limb or in the contralateral limb (Figure 3B). Such distant responses are more frequently observed in younger infants and likely reflect the high excitability and limited selectivity of early spinal and supraspinal circuits, consistent with developmental reductions in reflex responsiveness and in the incidence of mechanically evoked responses reported in infancy (156, 161, 162). As age increases, these widespread ipsilateral and contralateral responses become progressively less frequent (Figure 3B), indicating a developmental shift toward more differentiated and joint-specific motor activation.

The developmental profile of StR and ShR, and particularly the transition from widespread, easily elicited responses to more selective activation, has important diagnostic relevance: atypical patterns, such as absent or poorly modulated responses, or the persistence of broad ipsilateral or contralateral activation, may indicate impaired integration of proprioceptive input or altered maturation of spinal and descending pathways (163–165). Because PM-evoked responses probe circuits that underlie early tone regulation, postural control, and emerging voluntary movement, they may provide objective and clinically meaningful markers for identifying neuromotor dysfunction, especially in high-risk populations such as preterm or neurologically vulnerable infants (151). Combined with GMs and primitive reflex assessments, passive limb movements contribute to more accurate and timely detection of developmental alterations, providing an objective, age-sensitive assessment of muscle tone.

### 3.5 Development of early locomotor patterns and muscle coordination

**Figure 4:**
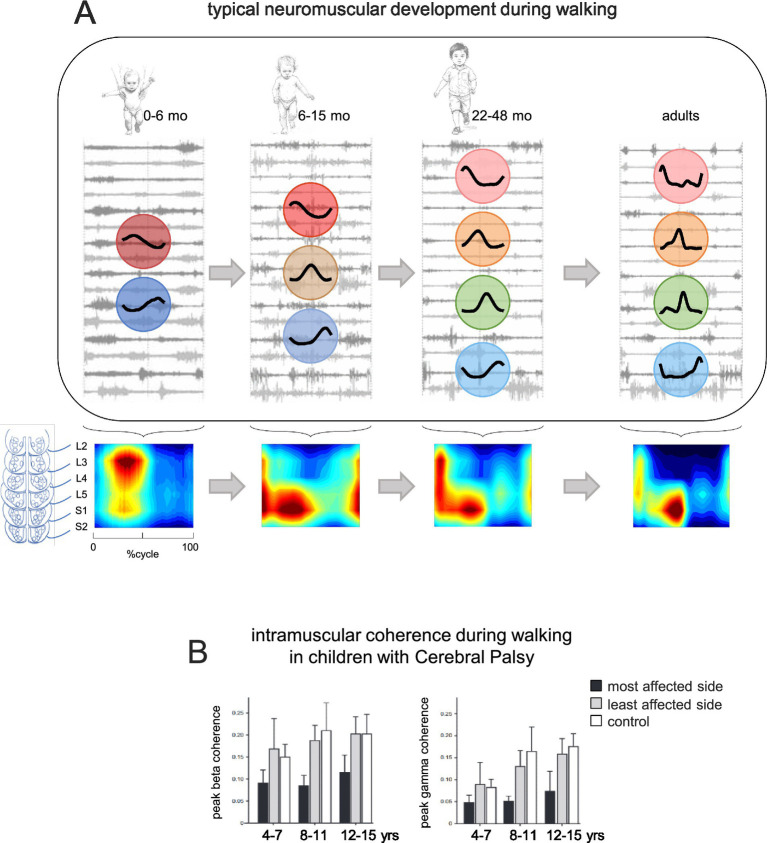
Locomotor modules development and neuromuscular activity. **(A)** Development of neuromuscular modules from the first days of life to adulthood. In neonates, motor output is organized in two basic modules. As locomotor control matures and independent walking emerges, module complexity progressively increases to four, reflecting the differentiation of proximal and distal flexor/extensor patterns and the overall refinement of motor coordination [redrawn from (29)]. Spinal maps of motoneuron (MN) activity in the lumbosacral segments (L2–S2), reconstructed by combining the averaged EMG envelopes of the ipsilateral leg with the anatomical locations of MN pools, are illustrated below and further emphasize developmental differences in neuromuscular organization [adapted from (28)]. **(B)** Neuromuscular (EMG–EMG) coherence during walking as indicator of locomotor impairments in children with cerebral palsy (CP). Peak coherence in the beta (15–25 Hz) and gamma (30–45 Hz) frequency bands for the tibialis anterior muscle is shown for three age groups (4–7, 8–11, and 12–15 years) in children with unilateral CP and in typically developing peers [from (172)].

Early locomotor-like behaviour provides critical insight into the maturation of neural circuits underlying movement and offers potential markers for identifying atypical motor development. One of the first observable manifestations of locomotor activity in neonates is the stepping reflex, consisting of alternating leg movements elicited by vertical suspension of the child with the feet contacting a surface. Kinematic analyses have shown that these early steps are quasi-rhythmic and coordinated, providing a framework for assessing the emergence of locomotor patterns (1, 2, 166). Surface EMG further reveals the neuromuscular organization underlying these movements. Neonatal stepping is characterized by simple alternating modulation of muscle activity across the step cycle, with coactivation of extensors during stance and flexors during swing (27, 29). A significant percentage of limb movements are bilaterally synchronous, rather than alternating (166–168). Despite apparent complexity, only two core muscle activation patterns (or modules), one flexor and one extensor, are sufficient to explain most of the observed activity (Figure 4A). These modules represent foundational locomotor primitives generated by spinal circuits, progressively refined and complemented during development (27, 28, 169). Compared with GMs, which engage multiple flexible synergies, neonatal stepping relies on just two consistent modules, reflecting a structured motor output shaped by both spinal pattern generators and sensory feedback. EMG activity also shows broad, overlapping bursts across lumbar and sacral motoneuron pools, indicative of an immature but coordinated spinal output that later becomes more temporally and spatially organized as locomotor control matures [Figure 4A; (28)].

Following the neonatal stepping reflex, infants gradually progress through a sequence of motor behaviours until the emergence of independent locomotion, a critical milestone in motor development both in typically developing (TD) children and in those with neuromotor disorders. In TD infants, the modular complexity of muscle activation progressively increases over the first year of life: stepping movements initially rely on two fundamental activation patterns, which gradually diversify and are refined as independent walking emerges (Figure 4A). This increase in the number of modules supports more flexible and adaptive control of gait, enabling precise coordination of posture, weight acceptance, and propulsion during unsupported walking (27, 28, 140). In children with neurodevelopmental disorders, such as CP, this maturation of spinal locomotor output is markedly delayed. While two core activation patterns are present during assisted or early unsupported stepping, the full complement of four basic muscle modules emerges only at the onset of independent walking, reflecting the delayed functional differentiation of proximal and distal extensor muscles. Prior to independent walking, children with CP show low-dimensional control and extensive coactivation across muscles, indicative of immature spinal locomotor circuitry (Avaltroni et al., under review). The delayed emergence of additional modules likely results from impaired corticospinal input and reduced ability to fractionate spinal motor patterns, which in TD children supports the addition of new synergies during the transition to independent walking [Figure 4A; (27, 29); Avaltroni et al., under review]. This pattern highlights a key principle in early motor development: low-dimensional, stereotyped control of stepping is a hallmark of immaturity, whereas the augmentation of modules allows for adaptive gait and the superimposition of voluntary movements. In CP, the preserved two-module structure prior to independent walking mirrors the limited variability observed in early spontaneous movements, suggesting a common mechanistic origin for motor impairment and reduced flexibility in gait control (170, 171).

In parallel with the modular organisation of muscle activity, EMG–EMG coherence during walking provides an additional measure of how shared neural input structures motoneuron activity. Similar to GMs, coherence reflects the integrity and synchrony of common synaptic drive and can reveal age-dependent maturation of motor networks. As illustrated in Figure 4B, in children with unilateral CP aged 4–15 years, coherence between synergistic muscles is often reduced or disorganised compared with typically developing peers, with attenuated peak values in the beta and gamma frequency bands during walking (172). These deficits are consistent with impaired corticospinal and spinal control and are associated with characteristic gait deviations, including abnormal activation of distal muscles such as the tibialis anterior (172, 173). Importantly, intensive locomotor interventions can partially restore coherence, reflecting plastic changes in descending motor pathways and improved motor control (174).

### 3.6 Future perspectives: AI-assisted clinical decision support

As neurodevelopmental research moves toward more quantitative and objective methodologies, the integration of Artificial Intelligence (AI) represents a key frontier for clinical translation. Recent advances have harnessed machine-learning and deep-learning frameworks to automate GMs analysis from video recordings (175–177). For example, marker-less pose-estimation combined with convolutional neural networks has achieved performance comparable to expert rates in classifying normal versus abnormal GMs (sensitivity ~76%–80%) (178). However, the future role of AI extends beyond simple automation; it lies in its potential to serve as a multimodal decision-support system that assists experts in improving diagnostic accuracy (179). By correlating patterns across the behavioural and neurophysiological domains discussed in this review, AI-driven models could offer a more holistic and granular risk stratification. Nevertheless, technical hurdles remain a significant barrier to clinical adoption, primarily the scarcity of large-scale, standardized paediatric datasets which limits the generalizability of current models. In this context, future efforts in Explainable AI (XAI) aim to provide clinicians with transparent rationales for diagnostic predictions, helping to reduce the subjectivity of traditional tools and supporting more personalized intervention strategies.

### 3.7 Interpersonal coordination

Toddlers often learn to walk by being led by the hand of a parent. This kind of haptic interaction plays a crucial role not just at the stage of the first independent steps by toddlers, but also during the whole childhood. A recent study highlighted the specific characteristics of the interaction forces exchanged by children while coordinating their locomotion with an adult or another child (180). These characteristics have clear distinguishing features relative to the same behaviour in dyads of interacting adults.

Interestingly, the cerebral mechanisms involved in interpersonal coordination appear to be evolutionarily conserved, as shown by comparing the neural activity recorded by functional magnetic resonance in human dyads and the neural activity recorded electrophysiologically in monkey’s dyads (181). Both monkeys and humans coordinate through adjustments based on proactive adaptation of motor planning and execution.

## 4 Clinical implications: from traditional to innovation-driven interventions

Early identification of neuromotor disorders has direct implications for therapeutic decision-making, as it enables the implementation of timely interventions during critical windows of neurodevelopment. For instance, locomotor experience and neural maturation are likely to interact reciprocally, given critical windows in supraspinal and spinal circuitry development and the need for early locomotor rehabilitation (182–185). Historically, the management of infants at risk of neurodevelopmental disorders has largely relied on supportive and reactive approaches, such as *Neurodevelopmental Treatment* (NDT), Vojta therapy, and conventional physiotherapy, often initiated only after the clinical manifestation of motor deficits (186–190). These strategies, while beneficial in improving functional outcomes, were typically introduced relatively late, limiting their potential impact on early brain maturation.

In recent years, a significant shift has occurred toward earlier, more targeted interventions, driven by a deeper understanding of neuroplasticity and the availability of reliable early markers, such as the assessment of spontaneous motor patterns (77, 191). This has paved the way for the implementation of early, activity-based, and goal-directed interventions, aimed at promoting motor learning and harnessing the plastic potential of the developing brain. Contemporary frameworks, including *Constraint-Induced Movement Therapy* (CIMT), *Hand-Arm Bimanual Intensive Therapy* (HABIT), and task-oriented training, emphasize active participation and repetition within enriched, family-centered environments (185, 192–195). In parallel, technological innovations, such as robotics-assisted rehabilitation, virtual reality-based environments, and wearable sensor systems, offer new opportunities for delivering intensive and individualized therapy therapy (196–198). Compared to traditional approaches, these strategies shift the focus from passive facilitation to active exploration and interaction with the environment, aligning more closely with principles of experience-dependent neuroplasticity. In this perspective, advances in early diagnosis not only improve prognostic accuracy but also enable more targeted intervention pathways, ultimately influencing long-term functional outcomes (78, 195).

Furthermore, new frontiers such as interpersonal coordination and haptic communication (180) represent fundamental areas for future study in children with CP. Given that sensorimotor impairments are often associated with cognitive deficits, understanding these communicative and social-motor dimensions may play a critical role in enhancing future rehabilitative strategies.

## 5 Conclusion

In conclusion, early spontaneous, passive, and locomotor-like movements provide a multi-level window into the maturation of sensorimotor circuits, capturing both neural and muscular organization. In particular, the assessment of GMs remains a cornerstone of early clinical prediction, offering a highly sensitive marker of neural integrity when combined with emerging quantitative and computational tools. This is a challenging field, characterized by high inter-individual variability and heterogeneity across different neuromotor disorders, which complicates the interpretation of early motor patterns. Future research integrating multi-modal, longitudinal assessments could help clarify the emergence of modular motor control and disentangle the contributions of corticospinal and subcortical networks. By combining behavioural, neurophysiological, and computational perspectives, such approaches offer a roadmap for translating mechanistic understanding into predictive biomarkers and targeted rehabilitation strategies. However, much work is still needed to translate specific motor outcomes in clinically validated primary or secondary endpoints to be used in diagnosis and rehabilitation.
